# How well is the electronic health record supporting the clinical tasks of hospital physicians? A survey of physicians at three Norwegian hospitals

**DOI:** 10.1186/s12913-019-4763-0

**Published:** 2019-12-04

**Authors:** Thomas Roger Schopf, Bente Nedrebø, Karl Ove Hufthammer, Inderjit Kaur Daphu, Hallvard Lærum

**Affiliations:** 1 Norwegian Centre for E-health Research, University Hospital of North-Norway, P.O. Box 35, 9038 Tromsø, Norway; 2 Norwegian Board of Health Supervision, P.O. Box 231 Skøyen, 0213 Oslo, Norway; 3 Centre for Clinical Research, Haukeland University Hospital, P.O. Box 1400, 5021 Bergen, Norway; 4 Section for e-Health, Department for Research and Development, Haukeland University Hospital, P.O. Box 1400, 5021 Bergen, Norway; 5 The Norwegian Directorate for e-health, P.O. Box 221 Skøyen, 0213 Oslo, Norway

**Keywords:** Electronic health record, Information and communication technology, Hospital physician, Clinical task, Clinical decision support

## Abstract

**Background:**

The electronic health record is expected to improve the quality and efficiency of health care. Many novel functionalities have been introduced in order to improve medical decision making and communication between health care personnel. There is however limited evidence on whether these new functionalities are useful. The aim of our study was to investigate how well the electronic health record system supports physicians in performing basic clinical tasks.

**Methods:**

Physicians of three prominent Norwegian hospitals participated in the survey. They were asked, in an online questionnaire, how well the hospital’s electronic health record system DIPS supported 49 clinical tasks as well as how satisfied they were with the system in general, including the technical performance. Two hundred and eight of 402 physicians (52%) submitted a completely answered questionnaire.

**Results:**

Seventy-two percent of the physicians had their work interrupted or delayed because the electronic health record hangs or crashes at least once a week, while 22% had experienced this problem daily. Fifty-three percent of the physicians indicated that the electronic health record is cumbersome to use and adds to their workload. The majority of physicians were satisfied with managing tests, e.g., requesting laboratory tests, reading test results and managing radiological investigations and electrocardiograms. Physicians were less satisfied with managing referrals. There was high satisfaction with some of the decision support functionalities available for prescribing drugs. This includes drug interaction alerts and drug allergy warnings, which are displayed automatically. However, physicians were less satisfied with other aspects of prescribing drugs, including getting an overview of the ongoing drug therapy.

**Conclusions:**

In the survey physicians asked for improvements of certain electronic health record functionalities like medication, clinical workflow support including planning and better overviews. In addition, there is apparently a need to focus on system stability, number of logins, reliability and better instructions on available electronic health record features. Considerable development is needed in current electronic health record systems to improve usefulness and satisfaction.

## Background

Information and communication technology (ICT) has become an essential part of the daily work of clinicians. Electronic health record (EHR) systems are expected to make health care services more efficient, reduce the workload of the clinician and prevent medical errors [1–5]. They can document diagnostic investigations and medical treatment, provide clinical decision support and facilitate communication between health care personnel. In addition, there is a growing demand to extract large data sets from the EHR for administrative reporting, clinical audits and research [5–7]. In the Nordic countries the EHR has become a standard tool for clinicians [8]. In recent years, improved EHR versions with an increasing number of functionalities have been implemented at Norwegian hospitals. According to the EHR vendors, these new releases are comprehensive and capable of providing all the features needed to support clinical work, patient administration and to facilitate research [9].

However, there is limited evidence on whether these new functionalities are beneficial [2, 10]. Positive effects are not guaranteed after implementing new EHR systems, and reports have indicated a variety of possible negative effects [1, 11, 12], especially regarding the usability of EHR systems [13]. For instance, Baumann et al. found that the time clinicians spend for documentation has increased after the introduction of EHR systems [14]. These experiences may also be related to non-clinical workload, e.g., documentation of coding and quality measures [13]. While many studies in the literature have focused on the implementation processes of EHR systems, there are few reports about fully adopted systems after longer periods of use [14–16]. A study by Kaipio et al. indicated that the usability of EHR functionalities in Finnish hospitals had not improved much during a 4 year period [16]. Both physicians and researchers have raised concerns that poor usability of the EHR may ultimately reduce the quality of clinical care [17, 18].

Research within cognitive informatics has increasingly been focusing on the use of ICT in clinical work environments, especially emphasizing the human computer interaction in a real world clinical context [19]. The “TURF” framework defines EHR usability in terms of how useful, usable and satisfying the EHR is in the clinical context [20]. Under “TURF”, usefulness refers to how well the system supports the users in accomplishing clinical tasks. A system is usable if it is easy to learn, intuitive to operate and with a low error rate. However, a system might be very usable, but still not useful if it does not support clinical tasks. A clinical task may refer to simple operations such as prescribing a drug or more complex processes such as gathering information for the discharge letter. Knowledge about usefulness is essential for improving deficiencies in the current systems, and for developing better EHR systems in the future. The aim of our study was to investigate the usefulness of EHR systems currently implemented in Norway. We were particularly interested in how well the clinical tasks were supported by EHR functionalities.

Since the technical infrastructure (e.g., computer processing power and speed) will influence the performance of the EHR, we also examined the perceived usefulness of the entire ICT environment. We sought to answer these research questions:

- How useful is the EHR?
- Which EHR functionalities are missing, and which need to be improved?
- What is the overall satisfaction with the EHR?
- How satisfied are physicians with the general ICT performance, e.g., system response time and reliability?

## Methods

### Setting

Several in- and outpatient clinics at three Norwegian hospitals participated in the study: Oslo University Hospital (OUH), Haukeland University Hospital (HUH) and Haraldsplass Deaconess Hospital (HDH). Each hospital had implemented the EHR system DIPS (DIPS ASA, Oslo) over the previous years (OUH in 2014, HUH and HDH in 2010). DIPS is one of the major EHR systems in Norway designed for clinical documentation, lab test management and prescription of drugs. All hospitals had the same version of DIPS. In addition to DIPS, there are several other independent ICT systems to help physicians in their clinical work – systems for radiological image archives, pathology reporting, etc.

### Data collection

A random selection of physicians at several clinical departments at the three hospitals was invited by email to participate in an online survey. While the head of each department provided the email addresses, all invitations (and reminders in case of no answer) were managed by the researchers. The departments were selected in such a way that the number of physicians from medical and surgical specialties could be balanced. The survey was conducted between November 2015 and September 2016. In case of no response, up to 10 reminders were sent.

### The questionnaire

The web-based tool LimeSurvey (LimeSurvey GmbH, Hamburg) was used for the online questionnaire. The tool automatically sent email reminders to participants and handled all data without disclosing any personal information (including IP-addresses). The questionnaire was developed by two of the authors (BN and HL) based on similar validated questionnaires used in previous studies, including a validated questionnaire from Lærum & Faxvaag (median kappa for use-related items 0.72 and for satisfaction-related items 0.62) [21, 22]. In a pilot trial (unpublished**)** in 2012, seven physicians from different clinical specialties reviewed the questionnaire, and their feedback was used to improve it. Our survey was thematically organized in the following three dimensions.

#### Dimension 1: technical performance

The physicians were asked about the technical performance of the EHR system, e.g., “How many times daily do you log in to access patient related information?” Further questions addressed computer response time, stability issues and the integration between different EHR applications.

#### Dimension 2: clinical tasks

**Fig. 1:**
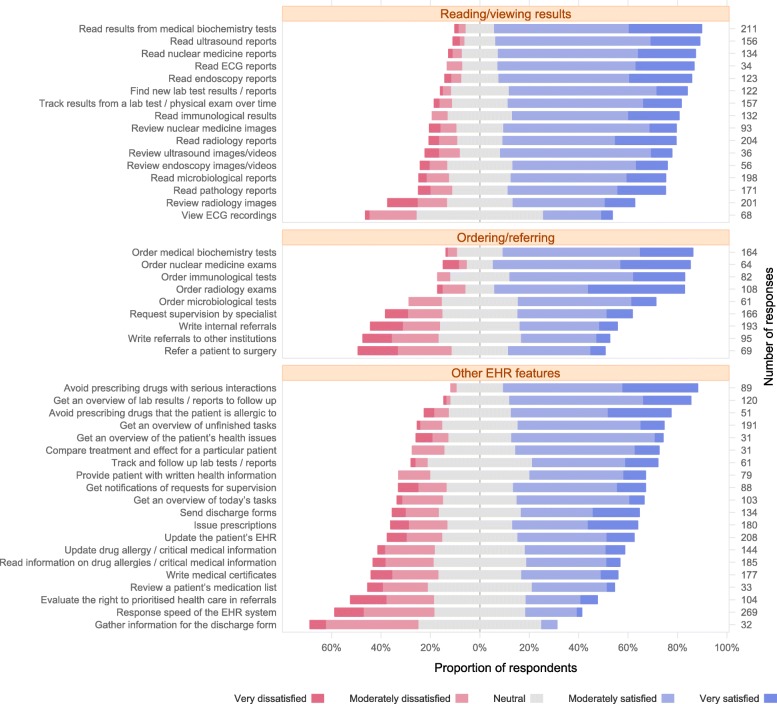
Satisfaction with functionalities. Respondents’ satisfaction with features of the EHR system. The questions used the phrasing ‘How satisfied are you with the support in DIPS for […]’, and are here abbreviated (and translated into English). The five response options ranged from ‘Very dissatisfied’ to ‘Very satisfied’, and the figure shows the percentage of respondents for each response option. The number of responses for each question is shown in the column to the right. Items with less than 30 responses are omitted

**Fig. 2:**
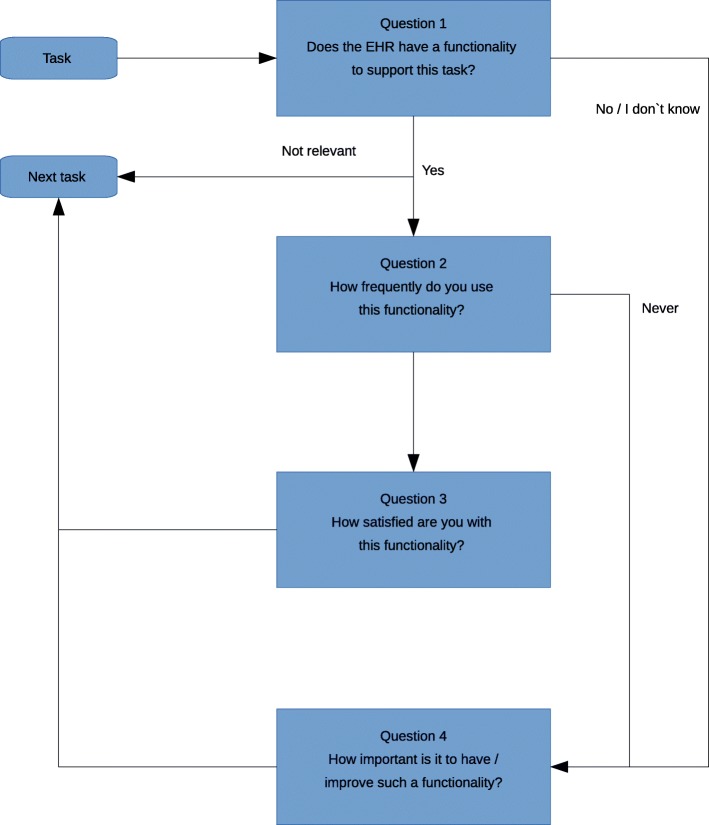
Flowchart. The flowchart displays the sequence of questions used in the survey

The physicians rated the compatibility of the clinical tasks with the functionality of the EHR. We examined 49 common clinical tasks (Fig. 1) regularly performed by physicians, along with the corresponding EHR functionalities designed to support these tasks. The tasks were selected based on previous studies [21, 23]. The questionnaire used branching logic to limit the total number of questions each physician had to answer (Fig. 2). Up to four questions were presented for each clinical task. First, each task was precisely defined. In question 1 in each four-question group, we asked if the EHR at the physician’s workplace had a functionality to support this clinical task. In addition to ‘Yes’ and ‘No’, the physician could also respond ‘Not relevant’ (e.g., if the task was never performed) and ‘I don’t know’. In question 2, we asked how frequently the physician used this EHR functionality in their daily work. In question 3, we asked how satisfied the physician was with the EHR functionality. If the physician responded ‘Not relevant’ in question 1, the remaining questions on the functionality were omitted. Instead, the four questions for the next clinical task were presented. If the physician responded ‘No’ or ‘I don’t know’ to question 1, a question on how important it would be to have this functionality was presented (question 4). If the answer to question 2 was ‘Never’ or ‘Almost never’, the physician was directed to a question asking how important it would be to *improve* such a functionality (question 4). The wording of question 4 would thus slightly change, depending on which answers were ticked in the previous questions.

Satisfaction was rated using 5-point Likert items (response options ‘Very dissatisfied’, ‘Moderately dissatisfied’, ‘Neutral’, ‘Moderately satisfied’, ‘Very satisfied’). The importance of improvements (question 4) was rated on a 4-point ordinal rating scale (response options ‘Not important’, ‘Slightly important’, ‘Important’, ‘Very important’).

#### Dimension 3: overall satisfaction

The physicians rated their agreement to statements regarding their overall satisfaction with the EHR system using 5-point Likert items (‘Completely disagree’, ‘Partially disagree’, ‘Neutral’, ‘Partially agree, ‘Completely agree’). Half of the statements were positively worded (i.e., ‘Completely agree’ indicated *satisfaction* with the EHR) and half of the statements were negatively worded (i.e., ‘Completely agree’ indicated *dissatisfaction* with the EHR).

### Analysis / statistical methods

For questions with categorical response options, we report counts and/or percentages for each possible response. For questions on the importance of having improvements in the EHR system, we scored the 4-point rating scale using the numerical values 0–3 and report the mean scores, along with 95% confidence intervals. To compute the confidence intervals, we used the percentile bootstrap, with 9999 bootstrap replications. We used R version 3.3.0–3.5.1 [24] to analyse the data and prepare the figures (except for the flowchart).

### Ethics

The Regional Committee for Medical and Health Research Ethics South East Norway has been consulted. According to national regulations ethics approval was not required because the study did not involve biomedical research and all data were anonymised.

## Results

The results are presented for all hospitals together. A total of 402 physicians were enrolled from three Norwegian hospitals, and 208 physicians (52%) submitted a completely answered questionnaire (Table 1). Physicians who reported that they had no patient contact or had been employed for less than 3 months, were excluded.

**Table 1:** Respondent demographics

	OUH	HUH + HDH	Sum
Hospital departments			
*Neurology*	81		81
*Ear-Nose-Throat (ENT)*	41		41
*Orthopaedics*	33		33
*Oncology*	73	40 + 0	113
*Internal medicine*		40 + 60	100
*Surgical dpt.*		40 + 21	61
Sum invited	228	201	429
Excluded	4	23	27
Total	224	178	402
Responses			
Complete responses	117 (52%)	91 (51%)	208 (52%)
Complete and partial responses	161 (72%)	141 (79%)	302 (75%)
Female respondents	47%	40%	44%

### Dimension 1

Only 21% of the physicians were satisfied or very satisfied with the processing speed of the EHR. Seventy-two percent of the physicians had their work interrupted or delayed because the EHR hanged or crashed at least once a week, while 22% had experienced this problem daily. Sixty-two percent of the physicians had to log in with a username and password more than 10 times per day, and 32% more than 20 times per day. Regarding the use of additional ICT systems, 58% reported that they were using more than one ICT system to access the patient data, and 10% were using more than three. For physicians using more than one ICT system, 82% had to look up the patient manually when logging in to two or more ICT systems, i.e., there was no functionality to automatically recognize which patient the physician was currently working on. Thirty-one percent responded that such a manual search was required with four or more ICT systems.

### Dimension 2

**Fig. 3:**
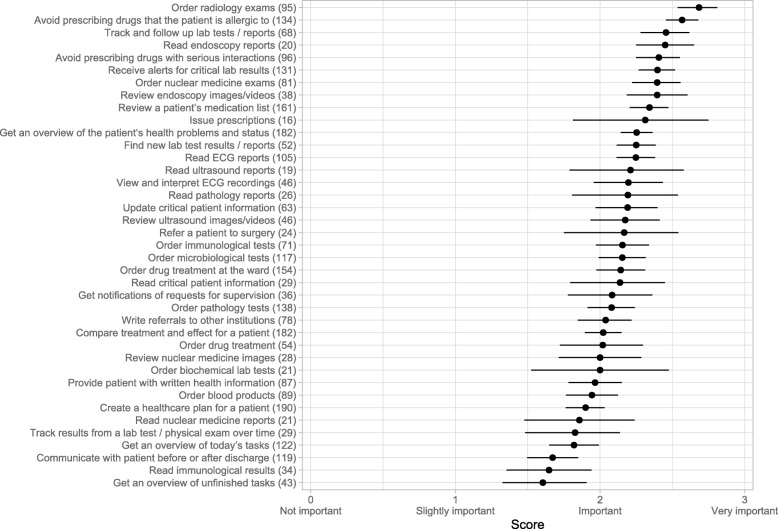
Priority of developing functionalities. Respondents’ view on the importance of developing / improving various features of the EHR system. The questions used the phrasing ‘How important is it to develop / improve […]’ (Fig. 1). The four response options ranged from ‘Not important’ to ‘Very important’, and are scored from 0 to 3. The circles correspond to the mean scores, and the lines to 95% confidence intervals. The number of respondents for each question is shown in parentheses after the question titles

Figures 1 and 3 show a selection of the results for dimension 2, arranged in functional categories corresponding to the clinical tasks.

### Dimension 3

**Fig. 4:**
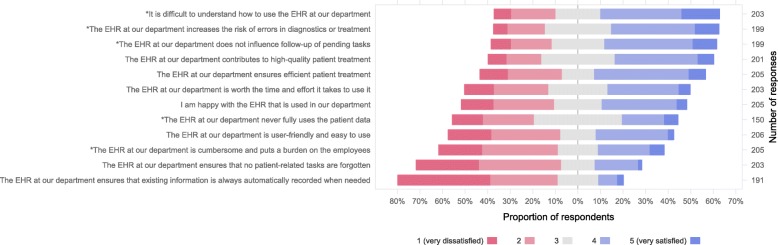
Overall satisfaction with the EHR. Respondents’ view on the overall quality of the EHR system. The question was introduced by the following text. ‘Lastly, we want to ask you a few questions on the overall quality of the EHR. What’s your opinion on the following statements?’ The five response options ranged from ‘Completely disagree’ to ‘Completely agree’. An equal number of positively and negatively worded (marked with * in the figure) statements was used. Here, the responses have been recoded so that high values always correspond to high satisfaction with the EHR

Figure 4 summarizes the results of dimension 3.

## Discussion

The majority of Norwegian doctors have been using EHR systems since the beginning of the twenty-first century [5, 8]. The EHR system in this study represents a fully adopted system, in contrary to previous usability studies in which the EHR systems were typically in a post-implementation setting demonstrating initial use experiences only [14–16].

Our findings indicated poor technical performance of the EHR. It appears that physicians often have to wait due to hanging computer systems. Based on our data, it is not possible to assess if these problems are caused by the EHR system itself, the operating system or the computer hardware. Yoo et al. studied requests for EHR improvements in a major Korean hospital and found no indications of poor ICT stability or hardware problems [25]. In contrast, Hyppönen et al. reported moderate satisfaction with system stability in Nordic hospitals [26].

Another time-consuming task is having to look up patients repeatedly when logging on other ICT systems. As different software companies may be involved, there might be no easy solution to this problem in the short run. However, health authorities in Norway have called for complete and easy access to all of the patient’s health data. As a potential result of standardization and integration of various data sources and computer applications, in the future, only a single EHR interface may be needed for clinicians to access all health data from both primary and secondary health care providers [5, 27].

We found several deficiencies in the compatibility of the EHR system with the most common clinical tasks. Few studies have examined the usefulness of EHR systems in terms of how well clinical tasks are supported. Kaipio and Viitanen have included clinical tasks in their studies, but in the questions presented to clinicians many similar functionalities were merged into groups [10, 16]. Novel EHR systems have complex functionalities which may consist of several minor “subfunctions” [5, 28]. For instance, instead of asking physicians about the overall usefulness of the drug management functionality, we have tried to elaborate on this by including all relevant EHR subfunctions available for managing drugs. There was high satisfaction with some of the decision support functionalities available for prescribing drugs (Fig. 1). This includes drug interaction alerts and drug allergy warnings, which are displayed automatically. However, physicians were less satisfied with other aspects of prescribing drugs, including getting an overview of the ongoing drug therapy. These findings indicate that a thorough examination of the different subfunctions of a complex functionality is needed in order to get a better understanding of how functionalities really work.

The majority of physicians were satisfied with managing tests, e.g., requesting and reading laboratory test results, radiological investigations and ECGs (Fig. 1). However, physicians were less satisfied with viewing radiological images or ECG recordings. Instead of accessing the final summary report, typically provided by specialists in radiology and cardiology, many physicians may find it useful to see the original image or ECG recording. This may be particularly relevant in emergencies, when there is no time to wait for the specialist’s interpretation, or when there is no specialist physically present at all, e.g., in small county hospitals.

Physicians were moderately satisfied with managing referrals, including incoming referrals as well as referrals of patients to other departments or hospitals. However, many physicians were dissatisfied with the functionality for writing and editing summary notes to primary health care, including discharge letters (Fig. 1).

We found that many items with high satisfaction scores apparently represent ‘simple’ functionalities, such as requesting and displaying lab results. There were only a few exceptions to this, e.g., the drug interaction and allergy warning alerts. Many basic and frequently used functionalities, such as managing sick notes or prescribing drugs, received only moderate scores (Fig. 1). Other examples include communication features for improving the interaction with other clinicians (e.g., discharge letters or referrals). In our opinion, important functionalities such as managing sick notes or prescribing drugs need urgent improvement. Since they represent specific and well-known procedures previously managed by simple paper forms, we are confident that neither complicated interface design work nor excessive computer processing power is needed to accomplish this.

Sixty-four percent of physicians correctly identified if the EHR had a functionality to support a certain clinical task (question 1). This may imply that the instructions given to physicians regarding the available features of the EHR are insufficient, as a considerable number of functionalities appears to be unknown. Similar findings have been reported by Price et al. [29].

Some of the overall scores in dimension 3 are low (Fig. 4). For instance, many physicians indicated that the EHR adds to their workload and may in fact be the cause of medical errors. There have been reports in the literature regarding concerns about safety and possible patient harm [1, 30–32]. Howe et al. reported that such adverse events might be due to a great variety of EHR usability issues, including data entry procedures and poor visual display of information [30].

Previously, the main purpose of patient health records was simply to document the work of clinicians, but the EHR is now expected to support the decision making process [1, 5, 18]. Modern decision support systems can potentially provide the clinician with all relevant previously recorded information needed to diagnose and provide medical treatment. The basic idea is to retrieve and process information automatically, and to make relevant data accessible to the clinician. The ultimate goal is to prevent medical errors, improve patient outcome and reduce the time the clinician has to spend searching for and recording information.

Sinsky has asked for a fundamental review of how the EHR is designed and integrated into the workflow of clinicians. She pointed out that even basic security tasks such as multiple log-in procedures may lead to increased workload and distract from clinical work [33]. In our opinion the findings in this study may support her views.

Limitations: The number of respondents in our study is limited. The results might be different in a larger study. In addition to DIPS, the physicians were using other independent ICT systems. We are aware that some of these systems are not the same at the 3 hospitals. Therefore some of the data might not be comparable. However, we analysed the data from each hospital separately and found no major differences. Since only physicians participated in our survey, we have no data to indicate the perceptions of other clinicians. Several professions use EHRs and their needs might differ from that of physicians. Also, we have little knowledge regarding users' expectations of the EHR. The usability issues identified in our study may be related to poor functionalities. However, we have not considered insufficient navigational design, e.g., if the clinician has to perform many mouse clicks or spend time scrolling down a huge document. As a consequence, the quality of that functionality may be perceived as poor. Finally, to our knowledge, the EHR systems studied are implemented in Norway only. The results may thus not be generalizable to EHR systems of other countries. Nevertheless, we believe that some common themes emerging from our study are of interest to the research community.

## Conclusion

In the survey physicians asked for improvements of many EHR functionalities. In addition, there is apparently a need to focus on system stability, number of logins, reliability, better instructions on available EHR features and improved integration between different clinical ICT systems and the EHR. Considerable development is needed in current EHR systems to improve usefulness and satisfaction.

## Data Availability

The datasets generated and analysed during the current study are available from the corresponding author on reasonable request.

## Abbreviations

EHR: Electronic health record
HDH: Haraldsplass Deaconess Hospital
HUH: Haukeland University Hospital
ICT: Information and communication technology
OUH: Oslo University Hospital
